# Structural equation model of affecting factors on elder abuse to patients under hemodialysis by family caregivers

**DOI:** 10.1186/s12877-021-02291-x

**Published:** 2021-06-12

**Authors:** Amaneh Mahmoudian, Abbas Shamsalinia, Atefeh Alipour, Zahra Fotoukian, Fatemeh Ghaffari

**Affiliations:** 1 Golestan University of Medical Sciences, Gorgan, Iran; 2 Nursing Care Research Center, Health Research Institute, Babol University of Medical Sciences, Shahid Motahari St, Ramsar, Mazandaran Ramsar, Iran; 3 Nursing Care Research Center, Health Research Institute, Babol University of Medical Sciences, Babol, Iran; 4 Imam Sajjad Hospital, Mazandaran University of Medical Sciences, Mazandaran Ramsar, Iran

**Keywords:** Elder abuse, Older adults, Family caregivers, Chronic kidney disease, End stage kidney disease, Hemodialysis

## Abstract

**Background:**

The objectives of the present study were to determine the prevalence of older adults with hemodialysis (HD) abuse by family caregivers and the factors affecting it.

**Method:**

This is a correlational-causal study, which is conducted in 2018 in Iran. The sample size was 367 in both groups (the older adults and their family caregivers). Data collection was done using an individual-social information questionnaire for the older adults under hemodialysis and their family caregivers, the questionnaire of elder abuse by family caregivers to the older people under hemodialysis, Zarit Burden Interview and the scale of instrumental activities of daily living (IADL). Data were analyzed by the structural equation model (SEM) method. The Fitness of proposed pattern was measured using the following indexes: chi-square/degree of freedom ratio (CMIN/DF), Normed Fit Index (NFI), comparative fit index (CFI), goodness of fit index (GFI), and standardized root mean squared residual (SRMR). The significant level in this study was considered p < 0.05.

**Results:**

The results of the present study showed that more than 70 % of the older adults suffer from elder abuse by family caregivers on average. The highest median elder abuse was related to emotional misbehavior (21.46 ± 6.09) and financial misbehavior (19.07 ± 5.33), respectively. Moderate care burden was experienced by 63.2 % of caregivers. The percentage of older women and men, who needed help with daily activities was 81.4 and 80.5 %, respectively. The results showed that the caregivers’ level of education and care burden with standard beta coefficient of -0.251 and 0.200 and the educational level of older adults and IADL with the best beta coefficient of -0.299 and − 0.234, had the highest regression effect on elder abuse respectively. According to the results, the model-fit indices of the hypothesized model was meet the criteria, with the NFI = 0.951, GFI = 0.970, CFI = 0.967, and SRMR = 0.041. The outcome was suitable for the recommended level, so the hypothetical model appeared to fit the data.

**Conclusions:**

The results of the present study showed that the prevalence of elder abuse by family caregivers among the older adults under hemodialysis is high. Providing psychological counseling can reduce the consequences of elder abuse.

**Supplementary Information:**

The online version contains supplementary material available at 10.1186/s12877-021-02291-x.

## Background

The global population of people aged 60 years old and older is expected to grow from 900 million in 2015 to almost 2 billion in 2050. Therefore, considering the aging population of many countries, the number of older adults who suffer from elder abuse is expected to increase. The world health organization (WHO) estimates that one in six older people aged 60 and older will experience elder abuse [1]. In a systematic review conducted by Ghiasi et al. (2018) in Iran, the total rate of elder abuse was reported to be between 14.7 and 87.8 % [2]. Molaei et al. (2017) also reported a 56.4 % prevalence of the subject among the Iranian older adults [3].

According to the centers for disease control and prevention (CDC), elder abuse is an intentional act or failure to act that causes or creates a risk of harm to an older adult or violates the human rights and reduces the life quality of an older adult (over 60 years) [4]. Human rights violations include physical, sexual, psychological and emotional misbehaviors, as well as financial and material abuse, abandonment, neglect and a serious lack of dignity and respect toward the older adult [5].

Although elder abuse may occur by HCPs, employees of long-term care centers for the older adults, or any other citizen, the abuse by family caregivers can have far-reaching consequences. Identifying and managing such abuses and the many challenges associated with it is a priority for public health and a major concern for health policymakers [6]. The increase in the prevalence of chronic and often disabling diseases among the older adults is associated with functional reduction in daily activities which lead to more dependence on HCPs and family caregivers [7]. Older people’s dependence on caregivers, along with poor health and chronic conditions, may expose them to abuse, neglect, and violence by family caregivers [8]. Today, due to policies of keeping the older adults at home, the number of older people receiving home care is increasing. In most societies, family members are responsible for caring for them. Caring problems, especially the problems of caring for an older adult, who has extensive care needs due to chronic illness, has increased the likelihood of violence and abuse by family caregivers [9, 10].

Findings of Heravi Karimou’s research (2011) showed that 25.9 % of the older adults have experienced at least one type of abuse by family caregivers. The highest prevalence of behavioral abuse was related to emotional neglect (17.4 %) and psychological abuse (17.2 %) and the lowest was related to rejection (3.7 %) and physical abuse (7.4 %) [11]. The results of a study by Rahimi et al. (2016) also showed that 62.8 % of the older adults have experienced caring negligence, 41 % mental abuse, 27.4 % physical abuse, 36.7 % financial misbehaviour, 48.6 % deprivation of authority, 25.6 % % rejection, 36.5 % financial negligence and 36 % have experienced emotional negligence [12]. Elder abuse in the family is defined as the imposition of pain and suffering on the older adults by family members, which may occur intentionally or unintentionally by committing an annoying act or leaving a necessary act intentionally or unintentionally, once or for a couple of times [13]. Elder abuse by family caregivers depends on a variety of factors, such as caregiver-related factors or patient-related factors. In their studies, a number of researchers have pointed to the effective factors of elder abuse. For instance, Yan et al. (2011) consider factors such as the number of days of living together and rejecting the help of domestic helper as well as the burden of care as predictors for elder abuse [14]. Rahimi et al. (2016) also considered age, number of children, amount of income and marital status as the effective factors in abuse and neglect of an older adult by family members [12].

Chronic kidney disease (CKD) and related therapies such as hemodialysis are one of the factors that may provide grounds for abuse in various dimensions [15]. The incidence of CKD has increased due to the increasing population of the older adults, the high prevalence of diabetes, hypertension and cardiovascular disease in the world [16].

Since the elder abuse can be associated with serious physical harms and long-term psychological consequences such as physical damages, loss of efficiency, social isolation, despair, hopelessness, depression, and reduced life, health and safety satisfaction in the older people [17]. Recognition of the influencing factors on this phenomenon can lead to a reduction in the possible serious consequences of it. However, despite the world health organization’s emphasis on international awareness on identifying and preventing elder abuse, the influencing factors for different groups with different backgrounds are still unknown. As the elder abuse by family caregivers is multifactorial and can depend on culture, nature of the disease, and the individual, social and clinical characteristics of the older adults and their family caregivers [18], it is therefore necessary to recognize the factors influencing on the phenomenon based on cultural contexts and individual backgrounds, so that intervention programs can be developed and implemented [5, 19].

In Iran, cultural barriers and the lack of support systems have prevented most elderly people, especially hemodialysis patients, from reporting cases of elder abuse to HCPs at the time of admission to clinical centers and during the treatment process. These barriers have made HCPs reluctant to investigate and report cases of elder abuse [20, 21]. Studies that examined the prevalence of elder abuse and its related factors in this group of Iranian older population were also not found during the literature review. Recognizing this problem and the factors influencing it can help HCPs to consider elder abuse and related factors in their care priorities and make evidence-based interventions.

### This study aimed to:

1.  Determining the prevalence of elder abuse to patients under hemodialysis by family caregivers and
2. Determining the effective factors in the elder abuse to patients under hemodialysis by family caregivers.

### Research questions include:

1. What is the prevalence of elder abuse among the patients under hemodialysis by family caregivers.
2. What are the effective factors in the elder abuse to patients under hemodialysis by family caregivers.

## Methods

### Design

This is a correlational-causal study conducted in 2018 in Iran.

### Sample

The study population was all the older people under hemodialysis who referred to medical centers in the western cities of Mazandaran province and the eastern part of Gilan province and their family caregivers.

The total number of hemodialysis patients in the east of Guilan and west of Mazandaran provinces was 563, of which 402 were 60 years old and older. There were 412 family caregivers. Convenience sampling was applied and the number of samples was determined using the following formula.


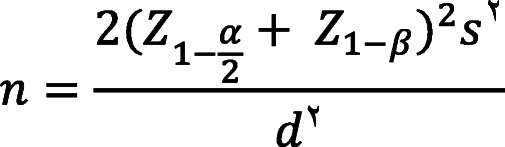


is the needed sample size, s = 24.5 and d = 0.2 s. The needed sample size was stimated to be 341 participants by accepting α = 0.05, ${Z}_{1-\frac{\alpha }{2}}$=1.96, 80 % of sample power and ${Z}_{1-\beta }=0.84$. Taking into account the 10 % probability of sample loss, the number of sample size for each group of hemodialysis older adults and their family caregivers was considered to be 370 people.

### Inclusion criteria

**For older adults: **being 60 and older, history of hemodialysis for at least one year, not having confirmed mental illness, lack of sensory disturbances such as blindness and deafness and receiving the score of 7 and above at the abbreviated mental test [19].

**For caregiver**: caring for an older adult under hemodialysis, being the patient’s relative, not caring another older adults and not having physical or mental illnesses (self-report).

### Exclusion criteria

Unwillingness for cooperation.

### Data collection

The research tool was completed through interviews for the elderly group, but self-administered by the family caregiver group. Data was collected by the following tools:

#### 1- Individual-social information questionnaire related to older people under hemodialysis

Age, sex, marital status, level of education, number of children, occupation, roommate, frequent hospitalization due to chronic illness, having other chronic diseases, duration of hemodialysis, being the head of family, ability of doing personal activities, level of need for daily care, member of the hemodialysis association, drug abuse, financial sufficiency and type of medical insurance.

#### 2- Individual-social information questionnaire related to family caregivers

Age, sex, marital status, level of education, medical education, being patient’s relative, length of care for the older adults, chronic illness, care for another patient, being the source of family income, place of residence, housing status, occupation, number of family members, living with the patient.

#### 3. The questionnaire of Elder Abuse to the patients under hemodialysis by Family Caregivers

This tool has been designed and psychometrically assessed by Mahmoudian et al. (2018). It has an acceptable internal validity (α = 0.98) for measuring the construct of elder abuse by family caregivers. It includes 57 items and 7 subscales including psychological misbehavior (6items), authority deprivation (7items) physical misbehaviours (2 items), financial misbehaviours (11 items), being abandoned (4 items), caring neglect (8 items) and emotional misbehavior (19 items). The tool is scored through a 4 point Likert scale with the options of never (1), sometimes (2), often (3) and always (4). Scores range between 1 and 228. Receiving the score of 1–75 indicates low severity of abuse, 76–152 average and 153–228 extreme severity of abuse [15].

#### 4- Zarit Burden Interview

This tool was designed by Zarit et al. (1988). It includes 22 items and three dimensions of role-playing stress, Intra-psychic stress, and Competencies and expectations. Caregiver’s responses are assessed through a five point Likert scale (never to always) and the scores range between 0 and 88. Getting a score of 0 to 20 showed little or no care burden, score of 21 to 40 a moderate, and the score of 41 to 88 a severe care burden [22, 23]. The validity of this tool was investigated and confirmed by Talebi et al. (2016). Its reliability is also confirmed by Cronbach’s alpha coefficient of 0.86 [24].

#### 5-Instrumental Activities of Daily Living (IADL)

This tool was first developed by Lawton & Brody (1969) and includes 8 dimensions of using of telephone, shopping, meal preparation, housekeeping, laundry, mode of transportation, medication management and money management for women. Women are scored on all 8 areas of function; historically, for men, the areas of food preparation, housekeeping, laundering are excluded. Independent (no help = 2), needs help (with a little help = 1) and dependent (cannot do that = 0) based on a 3 point likert scale is used for responding to the questions. A summary score ranges from 0 (low function, dependent) to 8 (high function, independent) for women and 0 through 5 for men. For women, the score of zero indicates a completely dependent situation, 1 to 15 needs for assistance, and the score of 16 indicates a completely independent situation. For men, the score of zero indicates a completely dependent situation, 1 to 9 needs for assistance, and the score of 10 indicates a completely independent situation [25]. The reliability of this tool was controlled by Mehraban et al. (2014). The results showed that the scale has an acceptable validity and the intra-class correlation coefficient of the two stages of the test with r = 0.993 is confirmed [26].

### Data Analysis

The data obtained from this study were analyzed using AMOS24, SPSS26 softwares and SEM method. SEM is a general and very powerful multivariate analysis technique of the multivariate regression family. More precisely, it is a general linear model, which allows the researcher to test a set of regression equations simultaneously and to Examine the relationships between different variables at the same time [27]. The fitness of the proposed model with data was measured using the chi-square/degree of freedom ratio (CMIN/DF), Normed Fit Index (NFI), comparative fit index (CFI), goodness of fit index (GFI), and Standardized Root Mean Squared Residual (SRMR). P-Value less than 0.05 was considered as significant level.

## Results

Of the 370 samples in the two groups of older people under hemodialysis and their family caregivers, three were excluded from the study due to their refusal to continue cooperation and ignoring a complete revision of the study’s tools. Finally, the data of 367 people in both groups were analyzed. The number of patients being 60–65 years old and under hemodialysis was found to be 39 % of the total 367. Males included 51.5 % of the samples, 32.2 % had a college degree and 67 % were married. Furthermore, 73.6 % of the samples had a history of recurrent hospitalization due to chronic disease (Table 1).

**Table 1:** Demographic characteristics of the older adults under hemodialysis experiencing different severities of elder abuse by family caregivers (*n*=367)

Variable	Subgroup	Number (%)	Severity of elder abuse by family caregivers		
			Low 1-75 Number (%)	Moderate 76-152 Number (%)	Extreme 153-228 Number (%)
**Age (years)**	60-65	143(39)	37(10.1)	103(28.1)	3(0.8)
	66-70	102(27.8)	26(7.1)	75(20.5)	0(0)
	71-75	65(7.7)	19(5.2)	46(12.6)	0(0)
	76-80	36(9.8)	7(1.9)	29(7.9)	0(0)
	81-85	16(4.4)	5(1.4)	11(3)	0(0)
	85<	5(1.4)	2(0.5)	3(0.8)	0(0)
**Sex**	Female	178(48.5)	50(13.7)	125(34.2)	3(0.8)
	Male	189(51.5)	46(12.6)	142(38.8)	0(0)
**Marital Status**	Single	5(1.4)	1(0.3)	4(1.1)	0(0)
	Married	246(67)	47(12.8)	196(53.6)	3(0.8)
	Divorced	11(3)	10(2.7)	1(0.3)	0(0)
	Widow	105(28.6)	38(10.4)	66(18)	0(0)
**Level of Education**	Illiterate	167(45.5)	19(5.2)	147(40.2)	1(0.3)
	Reading and writing	52(14.2)	14(3.8)	37(10.1)	1(0.3)
	Primary school	46(12.5)	11(3)	35(9.6)	0(0)
	High school	70(19.1)	31(8.5)	38(10.4)	1(0.3)
	University degree	32(8.7)	21(5.7)	10(2.7)	0 (0)
**Number of Children**	No child	28(7.6)	19(5.2)	9(2.5)	0 (0)
	1-3	92(25.1)	31(8.5)	58(15.8)	2(0.5)
	4-6	175(47.7)	41(11.2)	133(36.3)	1(0.3)
	7-9	61(16.6)	5(1.4)	56(15.3)	0 (0)
	More than 10	11(3)	0(0)	11(3)	0 (0)
**Occupation**	Unemployed	49(13.4)	2(0.5)	47(12.8)	0 (0)
	Farmer	37(10.1)	9(2.5)	28(7.7)	0 (0)
	Labor	20(5.4)	4(1.1)	16(4.4)	0(0)
	Housewife	105(28.6)	23(6.3)	79(21.6)	3(0.8)
	Employee	12(3.3)	7(1.9)	5(1.4)	0(0)
	Retired	73(19.9)	24(6.6)	48(13.1)	0(0)
	Self-employed	26(7.1)	13(3.6)	13(3.6)	0(0)
	Disabled	45(12.3)	14(3.8)	31(8.5)	0(0)
**Roommate**	Alone	50(13.6)	24(6.6)	25(6.8)	0(0)
	With spouse	121(33)	41(11.2)	80(21.9)	0(0)
	With children	81(22.1)	24(6.6)	57(15.6)	0(0)
	With spouse and children	111(30.2)	7(1.9)	100(27.3)	3(0.8)
	With others	4(1.1)	0(0)	4(1.1)	0(0)
**Frequent hospitalization due to chronic illnesses**	Yes	270(73.6)	74(20.2)	192(52.5)	3(0.8)
	No	97(26.4)	22(6)	75(20.5)	0(0)
**Type of insurance**	Social security	129(35.1)	20(5.5)	107(29.2)	2(0.5)
	Health services	149(40.6)	62(16.9)	86(23.5)	0(0)
	Rural inhabitants insurance	63 (17.2)	13(3.6)	50(13.7)	0(0)
	Others	26(7.1)	1(0.3)	24(6.6)	1(0.3)
**Experience of hemodialysis (months)**	24>	120(32.8)	15(4.1)	103(28.1)	2(0.5)
	24-48	109(29.5)	42(11.2)	67(18.3)	0(0)
	48<	138(37.7)	40(10.9)	97(26.5)	1(0.3)
**Experience of Drug abuse**	Yes	44(12)	19(5.2)	25(6.8)	0(0)
	No	323(88)	77(21)	242(66.1)	3(0.8)
**Financial Adequacy**	Adequate	41(11.2)	20(5.5)	21(5.7)	0(0)
	Average	168(45.8)	44(12)	122(33.3)	1(0.3)
	Low	78(21.3)	13(3.6)	63(17.2)	2(0.5)
	Not adequate	80(21.8)	19(5.2)	61(16.7)	0(0)
**Ability of doing personal activities**	Completely	15(4.1)	2(0.5)	13(13.6)	0(0)
	A lot	64(17.4)	16(4.4)	46(12.6)	2(0.5)
	Average	145(39.5)	51(13.9)	92(25.1)	1(0.3)
	Low	101(27.5)	23(6.3)	78(21.3)	0(0)
	Not much	42(11.4)	4(1.1)	38(10.4)	0(0)
**Need for daily care**	Very much	49(13.4)	5(1.4)	44(12)	0(0)
	A lot	115(31.3)	30(8.2)	85(23.2)	0(0)
	Low	121(33)	26(7.1)	93(25.4)	2(0.5)
	Not much	65(17.7)	31(8.5)	32(8.7)	1(0.3)
	Not at all	17(4.6)	4(1.1)	13(3.6)	0(0)
**Having other chronic illnesses**	Yes	230(62.7)	63(17.2)	166(45.4)	1(0.3)
	No	137(37.3)	33(9)	101(27.6)	2(0.5)
**Head of family**	Yes	202(55)	56(15.3)	144(39.3)	1(0.3)
	No	165(45)	40(10.9)	123(33.6)	2(0.5)
**Membership in hemodialysis association**	Yes	297(80.9)	91(24.9)	204(55.7)	1(0.3)
	No	70(19.1)	5(1.4)	63(17.2)	2(0.5)

 The results showed that the mean and standard deviation of the age of family caregivers was 13.94 ± 49.85 years with a minimum of 18 and a maximum of 85 years. Female caregivers accounted for 64.9 % of the total caregivers and 79.8 % were married (Table 2).

**Table 2:** Individual characteristics of family caregivers by severity of care burden (*n*=367)

Variable	Subgroup	Number (%)	Severity of Care Burden		
			Low 0-20 Number (%)	Moderate 21-40 Number (%)	Extreme 41-88 Number (%)
**Age (years)**	30>	22(6)	13(3.5)	4(1.1)	5(1.4)
	50-30	174(47.4)	71(19.3)	70(19.1)	33(9)
	70-51	150(40/9)	57(15.5)	60(16.3)	33(9)
	71<	21(5.7)	11(3)	8(2.2)	2(0.5)
**Sex**	Female	238(64.9)	58(15.8)	44(12)	27(7.4)
	Male	129(35.1)	94(25.6)	98(26.7)	46(12.5)
**Marital status**	Single	49(13.4)	21(5.7)	18(4.9)	10(2.7)
	Married	293(79.8)	122(33.2)	118(32.2)	53(14.4)
	Divorced	14(3.8)	3(0.8)	5(1.4)	6(1.6)
	Widow	11(3)	6(1.6)	1(0.3)	4(1.1)
**Level of Education**	Illiterate	81(22.1)	30(8.2)	33(9)	18(4.9)
	Primary School	74(20.2)	31(8.4)	28(7.6)	15(4.1)
	High School	118(32.2)	40(10.9)	46(12.5)	32(8.7)
	University Degree	94(25.6)	51(13.9)	35(9.5)	8(2.2)
**Medical Sciences-related Education**	Yes	41(11.2)	21(5.7)	14(3.8)	6(1.6)
	No	326(88.8)	131(35.7)	128(34.9)	67(18.3)
**Relationship with the Patient**	Father	2(5)	1(0.3)	1(0.3)	0(0)
	Mother	10(2.7)	8(2.2)	1(0.3)	1(0.3)
	Spouse	143(39)	59(16.1)	64(17.4)	20(5.4)
	Sister	6(1.6)	3(0.8)	1(0.3)	2(0.5)
	Brother	3(0.8)	3(0.8)	0(0)	0(0)
	Child	166(45.2)	57(15.5)	63(17.2)	46(12.5)
	Others	37(10.1)	21(5.7)	12(3.3)	4(1.1)
**Duration of Care (months)**	24>	128(34.9)	48(13.1)	58(15.8)	22(6)
	24-48	92(25.1)	18(4.9)	61(16.6)	13(3.5)
	48<	147(40.1)	56(15.3)	73(19.9)	18(4.9)
**Caring another Patient?**	Yes	29(7.9)	6(1.6)	18(4.9)	5(1.4)
	No	338(92.1)	146(39.8)	124(33.8)	68(18.5)
**Having a Chronic disease**	Yes	94(25.6)	36(9.8)	33(9)	25(6.8)
	No	273(74.4)	116(31.6)	109(29.7)	48(13.1)
**Residence**	Urban	222(60)	107(29.2)	72(19.6)	41(11.2)
	Rural	145(40)	44(12)	69(18.8)	30(8.2)
**Housing Status**	Renting	63(17.2)	22(6)	22(6)	19(5.2)
	Owning	304(82.8)	130(35.4)	120(32.7)	54(14.7)
**Occupation**	Unemployed	30(8.2)	6(1.6)	15(4.1)	9(2.5)
	Farmer	26(7.1)	10(2.7)	11(3)	5(1.4)
	Housekeeper	145(39.5)	58(15.8)	58(15.8)	29(7.9)
	Labor	21(5.7)	3(0.8)	9(2.5)	9(2.5)
	Employee	36(9.8)	18(4.9)	11(3)	7(1.9)
	Self-Employed	49(13.4)	26(7.1)	15(4.1)	8(2.2)
	Retired	47(12.8)	22(6)	19(5.2)	6(1.6)
	Student	13(3.5)	9(2.5)	4(1.1)	0(0)
**Being the family income source**	Yes	155(42)	61(16.6)	62(16.9)	29(7.9)
	No	212(58)	87(23.7)	80(21.8)	43(11.7)
**Number of family members**	<3	137(37.3)	69(18.8)	49(13.4)	19(5.2)
	4-3	163(44.4)	64(17.4)	60(16.3)	39(10.6)
	>4	67(18.3)	19(5.2)	33(9)	15(4.1)
**Living with the patient**	Yes	276(75.2)	105(28.6)	114(31.1)	57(15.5)
	No	91(24.9)	47(12.8)	28(7.6)	16(4.4)

The results showed that the average total score of elder abuse by family caregivers was 19.75 ± 87.89. The mean total score of abuse severity considering the three levels of low (1–75), moderate (76–152) and extreme (153–228) was 60.53 ± 5.14, 96.2 ± 9.84 and 169.66 ± 22.30 respectively. The prevalence of elderly abuse by caregivers is presented in Table 3 according to the severity of the abuse. The results showed that the highest prevalence of elder abuse was related to moderate severity (70 %).

**Table 3:** Number and percentage of scores of elder abuse by caregivers and its aspects by the severity of elder abuse (*n* = 367)

Elder abuse by caregivers and its aspects	Mean ± SD	severity of elder abuse by caregivers		
		**Low** **Number (%)**	**Moderate** **Number (%)**	**Extreme** **Number (%)**
**Psychological Misbehavior**	10.86 ± 3.07	71(19.2)	245(66.8)	51(14)
**Authority Deprivation**	12.70 ± 3.23	60(16.3)	263(71.7)	44(12)
**Physical Misbehavior**	3.49 ± 1.08	103(28.1)	247(67.3)	17(4.6)
**Financial Misbehavior**	19.07 ± 5.33	94(25.6)	242(65.9)	31(8.5)
**Being Abandoned**	7.15 ± 2.14	91(24.8)	234(63.8)	42(11.4)
**Caring Neglect**	13.06 ± 4.40	79(21.5)	262(71.4)	26(7.1)
**Emotional Misbehavior**	21.46 ± 6.09	254(69.2)	97(26.4)	16(4.4)
**Total**	87.89 ± 19.75	96(26.2)	257(70)	14(3.8)

The results of the present study showed that the mean total score of care burden was 48.63 ± 16.74. The mean of the total score for the three severity levels of low (0–20), moderate (21–40) and extreme (41–88) were (11.35 ± 5.59), (29.28 ± 5.67) and (53.20 ± 8.61). Also, the highest mean of care burden (22.61 ± 8.75) was related to the role-related stress aspect. Moderate care burden was experienced by 63.2 % of caregivers (Table 4).

**Table 4:** Average, standard deviation and frequency distribution of care burden severity and its aspects by levels

Care burden and its aspects	Mean ± SD	Severity of care burden		
		**Low** **Number (%)**	**Moderate** **Number (%)**	**Extreme** **Number (%)**
**Role Related stress**	22.61 ± 8.75	109(29.7)	226(61.6)	32(8.7)
**Intra-Psychic Stress**	13.41 ± 5.86	96(26.2)	221(60.2)	50(13.6)
**Competencies & Expectations**	12.69 ± 4.11	101(27.5)	226(61.6)	40(10.9)
**Total**	48.63 ± 16.74	94(25.6)	232(63.2)	41(11.2)

The results showed that the mean total IADL scores for men and women were 7.68 ± 4.85 and 6.66 ± 3.62, respectively. The highest mean scores in older men and women were 1.28 ± 0.74 and 1.42 ± 0.66, respectively, for medication management aspect. The percentage of older women and men, who needed help with daily activities was 81.4 and 80.5 %, respectively (Table 5).

**Table 5:** Mean, standard deviation and frequency distribution of IADL and its dimensions by sex

IADL	Sex		Mean ± SD
**Using of Telephone**	Female		1.19 ± 0.73
	Male		1.37 ± 0.69
**Shopping**	Female		0.89 ± 0.81
	Male		1.08 ± 0.77
**Meal Preparation**	Female		0.96 ± 0.80
	Male		0
**Housekeeping**	Female		0.85 ± 0.72
	Male		0
**Laundry**	Female		0.80 ± 0.81
	Male		0
**Mode of Transportation**	Female		0.96 ± 0.79
	Male		0.96 ± 0.85
**Medication Management**	Female		1.28 ± 0.74
	Male		1.42 ± 0.66
**Money Management**	Female		0.74 ± 0.85
	Male		1.07 ± 0.80
**Total**	Female	Number of completely dependent (0) cases (%)	11(6.2)
		Number of cases needing help (1–15) (%)	145(81.4)
		Completely independent (16) (%)	22(12.4)
		total score	7.68 ± 4.85
	Male	Number of completely dependent (0) cases (%)	9(4.7)
		Number of cases needing help (1–9) (%)	152(80.5)
		Completely independent (10) (%)	28(14.7)
		Total score	6.66 ± 3.62

The results of Pearson’s correlation test showed that there was a significant negative relationship between IADL with care burden (*r*=-0.188, *P*-value < 0.01) and elder abuse (*r*=-0.113, *P*-value < 0.05). There was also a significant positive relationship between care burden and elder abuse (*r* = 0.285, *P*-value < 0.01). Stepwise method multiple regression was used to investigate the simultaneous effect of variables related to family caregivers and affecting elder abuse. The categorical variables are graded as follows: level of education [illiterate = 0 literate = 1], marital status [single = 0 married = 1], having a chronic disease [no = 0 yes = 1], medical sciences- related education [no = 0 yes = 1], financial adequacy [medium down = 0 up = 1], ability of doing personal activities [medium down = 0 high = 1], roommate [with spouse and children = 0 other = 1].

 Independent variables (demographic, clinical, and care pressure variables) were entered in model 6. The level of education entered the first model, explaining 27 % of the changes in elderly abuse by family caregivers. Also, in models 2 to 6, the variables of care burden, medical sciences-related education, chronic diseases, number of family members and marital status were entered, respectively, and finally explained 43 % of changes in the subject. These variables have been also able to make significant predictions for elder abuse by family caregivers. The results showed that the level of education and care burden with the standard beta coefficient of -0.251 and 0.200, respectively, have the highest regression effect on elder abuse by family caregivers (Table 6).

**Table 6:** The final model (sixth model) of regression of the effect of independent variables (related to family caregivers) on the dependent variable (elder abuse)

Model	Unstandardized Coefficients B	Std. Error	Standardized Coefficients Beta	t	Sig
**Constant**	68.626	9.141		7.508	0.000
**Level of education**	-4.540	0.927	-0.251	-4.997	0.000
**Care burden**	0.236	0.058	0.200	4.094	0.000
**Medical sciences-related education**	8.938	3.128	0.143	2.857	0.005
**Having chronic diseases**	5.380	2.188	0.119	2.459	0.014
**Number of family members**	1.796	0.744	0.118	2.415	0.016
**Marital status**	-3.718	1.786	-0.102	-2.082	0.038
**The summary of the 6th model**	,F = 48.105 P < 0.001		R-square = 0.496	adjusted- R-square = 0.428	

Stepwise Method multiple regression was used to investigate the simultaneous effect of variables related to older people under hemodialysis affecting elder abuse by family caregivers. Independent variables (demographic and clinical variables for the older adults and IADL) were entered in 8 models. The level of education entered the first model, explaining 34 % of the changes in elder abuse by family caregivers. Also, in models 2 to 8, IADL variables, with whom the older adult lives, ability of doing personal activities, financial adequacy, number of children, having other chronic diseases and age entered the model, which all explained 52 % of the changes to the subject. These variables had also the potential to provide significant predictions for elder abuse by caregivers. The results showed that the level of education of the older adults and IADL with the standard beta coefficient of -0.299 and − 0.234, respectively, had the highest regression effect on the elder abuse (Table 7).

**Table 7:** The final regression model (8th model) of the effect of independent variables (related to the older adult) on the dependent variable (elder abuse)

Model	Unstandardized Coefficients B	Std.Error	Standardized Coefficients Beta	t	Sig
**Constant**	66.848	7.817		8.551	0.000
**Level of education**	-4.160	0.720	-0.299	-5.780	0.000
**IADL**	-4.283	0.833	-0.234	-5.143	0.000
**With whom the older adult lives**	8.525	1.806	0.211	4.720	0.000
**Ability of doing daily activities**	-2.646	0.986	-0.135	-2.683	0.008
**Financial adequacy**	2.568	0.978	0.124	2.627	0.009
**Number of children**	3.433	1.088	0.157	3.156	0.002
**Having other chronic diseases**	4.740	1.901	0.116	2.493	0.013
**Age**	-1.648	0.786	-0.104	-2.098	0.037
**The summary of the 6th model**	F = 62.541 0.001, P<		0R-square = 0.559	adjusted- R-square = 0.517	

**Fig. 1:**
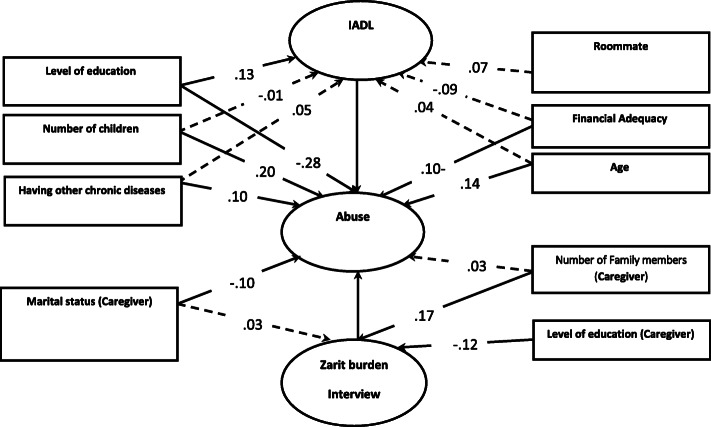
Structural Equation Modeling Results: The Relationship between Care burden and IADL with elder abuse by Family Caregivers Based on Demographic and Clinical Variables

In this study, SEM method was used to test the proposed model (Model 1) and the effect of independent demographic and clinical variables of both groups (Fig. 1) on the relationship between care burden and IADL with elder abuse.

### The normal distribution of data, outliers and missing data

To examine the research hypotheses before using the SEM method, the assumptions of this method were examined. For this purpose, single-variable and multi-variable data distribution were examined separately for natural distribution and discarded data. The existence of multivariate scatter data using Mahalanobis d-squared method (*P* < 0.001) and normal multivariate distribution using Mardia coefficient (above 20) were investigated [28]. The results showed that the normal single- and multivariate-distribution were normal.

Then, the fitness of the proposed model was evaluated based on the fitness indicators introduced in Table 8. Given that the value of CMIN/DF is less than 5 [29] and the value of SRMR is less than 0.1 [30], the fitness of the proposed model is confirmed. To improve the fitness of the proposed model, the model was modified in the next step by drawing a correlation between the errors.

**Table 8:** Fit indices of the primary and modified model

	GFI	NFI	CFI	SRMR	CMIN/DF	P-Value	Df	CMIN
**Primary model**	0.841	0.887	0.897	0.084	4.42	< 0.001	134	592.362
**Modified model**	0.970	0.951	0.967	0.041	2.83	< 0.001	126	357.593

Abbreviations; *CMIN/DF* Chi-square/degree-of-freedom ratio; *SRMR* Standardized Root Mean Squared Residual; *GFI* Goodness of Fit Index; *NFI* Normed Fit Index; *CFI* Comparative Fit Index. Fit indices: CFI, NFI, GFI (> 0.9), SRMR (< 0.05 good, 0.05–0.08 accept), CMIN/DF (< 3 good, < 5 acceptable)

The results showed that the two variables of care burden and IADL explained 43 % of the changes in elder abuse by family caregivers in the model. Standard regression coefficients showed that there was a strong negative relationship between IADL and elder abuse by family caregivers. Therefore, the greater the independence of the older adults, the lower the rate of elder abuse abuse (β = 0.12). There is also a positive and strong relationship between care burden and elderly abuse by family caregivers. As the care burden increases, the abuse increases (β = 0.20) (Model 1).

The structural function model after data fit with the assumed pattern is shown in Fig. 1 according to the demographic and clinical variables related to the older adults and related to the family caregiver. The results showed that the demographic and clinical variables related to the older adults, with whom the older adult lives (*r* = 0.29, *P*-value < 0.001), age (*r* = 0.14, *P*-value < 0.05), other chronic diseases (*r* = 0.10, *P*-value < 0.05) and the number of children (*r* = 0.20, *P*-value < 0.001) have a positive effect on the elder abuse respectively. However, the level of education (*r* = -0.28, *P*-value < 0.001) and financial adequacy (*r* = -0.10, *P*-value < 0.05) have negative effects. Therefore, the higher the level of education and financial independence of the older adults, the more we will see a decrease in the elder abuse by family caregivers. Also, demographic variables related to family caregiver (*r*=-0.19, *P*-value < 0.05) and marital status (*r*=-0.68, *P*-value < 0.001) have a negative effect on the subject, respectively. Therefore, the higher the level of caregiver’s education, the lower the rate of elderly abuse. But there is no significant relationship between the number of family members caring for the older adults and the elder abuse.

## Discussion

The objectives of this study were to determine the prevalence of elder abuse by family caregivers among older patients under hemodialysis and the factors affecting it. Overall, the results showed that more than half of the older adults under hemodialysis suffered from moderate abuse by their family caregivers, and patient’s level of education, IADL scores, caregivers’ level of education and his/her marital status had the highest regression effect on the elder abuse. Different information is available on the prevalence of elder abuse by family caregivers. For example, in the study of Orfila et al. (2018), the prevalence of the subject was 33.4 % [18] but the same number at the American studies is reported to be 5–10 % [31–33]. Manouchehri et al.‘s (2008) study also found that more than 87 % of respondents were at least once abused by family caregivers [34]. Such differences can be attributed to different research approaches, target groups, and data collection tools.

The highest median elder abuse was related to the emotional aspect. Negative reactions from family caregivers to the symptoms and consequences of CKD or related therapies such as hemodialysis, and expressing disgust as touching the central venous catheter or arterial-venous fistula, or feeling ashamed and therefore not accompanying the older adults in public places due to disease-related appearance changes, and ignoring physical contact with the older adults for fear of contagious disease are among the cases of emotional elder abuse by family caregivers. The high level of emotional abuse can be due to the ignorance of caregivers about how to establish respectful relationships and maintain human dignity with the older adults. The results of other studies showed that the highest score of elder abuse was related to the mental aspect. In Manouchehr et al.‘s (2008) research, emotional abuse and negligence have been the most common forms of abuse [34]. In Nouri et al.‘s (2013) study, 34.8 % of the older adults experienced emotional negligence [35].

The results showed that the level of education of the older adults and IADL had the highest regression effect on elder abuse. This means that with a unit increase in the level of education of the older adults and IADL scores, 0.251 and 0.234 units of decrease are observed in elder abuse, respectively. A high level of education in the older adults may cause them to be able to take better care of themselves and put less pressure on their caregivers. This finding is inconsistent with the results of a study by Orfila et al. (2018) [18]. Decreased energy levels, frequent need for hemodialysis and associated health problems, feelings of depression, and inability to perform normal daily activities, all affect the patient and disrupt his normal life and increase his dependence on family caregivers [24]. When the older adults become dependent on family caregivers for a variety of reasons, his self-care and self-management abilities get limited. In fact, time-consuming and tedious activities such as transporting the older adults to dialysis centers, caring for the older adults after dialysis, controlling blood pressure, injecting insulin in cases where the patient has diabetes, controlling the proper and timely use of oral medications, Preparing and supervising the nutrition of the older adults and helping him with many of the daily activities of his life, such as bathing, making food, shopping, etc., will be among the duties of family caregivers. In most cases, these tasks and associating them with tasks related to the personal and family life of caregivers imposes a lot of pressure on them, which sometimes leads to misbehavior with the older adults due to lack of social support and support from other family members[24, 26]. The results of a study by Dastyar et al. (2020) showed that there is a significant relationship between the need for care and the suffering of care. In a way, the more the patient needs care, the more the caregiver suffers. This suggests that the worse the functional level of patients and the greater their need and dependence on caregivers, the greater the suffering caused by this dependence in caregivers and more possibility of negligence [36].

The results showed that 63.2 % of family caregivers experienced moderate care burden. The results of the present study showed that there is a significant relationship between care burden and elder abuse. A study by Iborra et al. (2008) also showed that the negative effects of caring for dependent relatives such as caring burden, stress, mood disorders, social isolation and caregiver’s personality are effective factors in elder abuse by family caregivers [37]. The results showed that the level of caregiver’s education and care burden had the highest regression effect on elder abuse by family caregivers. The results of a study by Dastyar et al. (2020) showed that caregivers who had a higher level of education and were employed reported less care suffering than caregivers with lower education and unemployed [36]. The results of a study by Orfila et al. (2018) showed that the most relevant risk factors for elder abuse by family caregivers were the care burden, caregiver’s anxiety, caregiver perception of aggressive behavior in the care recipient, and bad previous relationship respectively [18].

 The results of the present study showed that the demographic variables related to family caregivers, respectively, the level of caregiver education and marital status, have a negative effect on elder abuse. Therefore, the higher the level of caregiver education, the lower the possibility of abuse. A higher level of education is likely to increase understanding of the conditions and needs of hemodialysis patients and increase the financial independence of the caregiver, thus making it less difficult to provide care services to older people. Married caregivers may also be more tolerant of the burden of care due to their higher sense of responsibility to their parents than to single people. The level of education in the results of the studies of Dastyar et al. (2020) [36] and Ashghali Farahani et al. (2016) [38] is also a factor in predicting the care burden and ultimately elder abuse by family caregivers.

The limitations of this study include the following: Due to the prevailing culture in Iranian society regarding the preservation of the dignity of the family and children, it may have not been possible for the patients to respond to the items freely. Convenience sampling method was used in this study. This method will impair the generalizability of the results. The study’s target group was older adults under hemodialysis and their family caregivers. Due to the nature of the disease, the participating patients may have experienced different abusing severities in different dimensions rather than the older people with other chronic diseases, so the results can only be generalized to this group of older adults and their family caregivers. The study used the elder abuse by family caregivers’ questionnaire to investigate the elder abuse and its dimensions. The data collected with this tool cannot confirm whether or not the abuse actually occurred. However, we consider it the best tool available to achieve the goal of our study because it has acceptable validity and reliability for measuring the structure we want. Understanding elder abuse by family caregivers varies from culture to culture, so generalizing the results may impair them. Given the self-reported nature of the abuse questionnaire, the likelihood of the older adults feeling guilty about talking about the subject and the problems they endure can affect the prevalence and severity of the phenomenon.

## Conclusions

The results of the present study showed that the prevalence of elder abuse by family caregivers among the older adults under hemodialysis is high.

Health and social policymakers can reduce the prevalence of elder abuse by the application of different programs such as requiring the screening of elder abuse, raising people’s awareness through media, mandatory reporting of abuse by healthcare professionals (HCPs), training programs for older adults, their family caregivers, and HCPs to identify the phenomenon of elder abuse, factors affecting it and how to deal with it. implementation of educational programs with the purpose of increasing self-confidence of the older people and development of support systems, which aim to reduce the prevalence of elder abuse and manage the effective factors in it, can be also listed as the aforementioned programs. Furthermore, providing psychological counseling, especially when the patient is referred to a medical center for hemodialysis, can reduce the consequences of elder abuse. The best time to train caregivers is when they are with the older adults in medical centers and hemodialysis departments. HCPs can develop and implement training and counseling programs to achieve the goal of reducing elder abuse at a time when the older adults are hospitalized for at least 3 to 4 h.

The results of the present study showed that one of the effective factors in elder abuse is the care burden. In the first step, intervention programs should be developed to reduce the care burden of family caregivers. Community support services need to be designed so that family caregivers can take advantage of the care they receive. Psychological interventions for family caregivers, such as participating in community support groups, getting help from formal caregivers, and getting help from other family members may reduce their care burden.

The results of the study showed that the dependence of the older adults on caregivers is associated with an increase in elder abuse cases. Primary prevention activities should be developed continuously and based on the community and the family in order to reduce the risk factors for the elder abuse. Interventions such as training on how to care for the older adults, financial assistance for cases with complete dependence through centers such as state welfare organization and adequate social support for caregivers may reduce the factors affecting the elder abuse.

The strengths of this study include the following: The results of this study, due to the high number of samples, provide a relatively broad view of the prevalence and factors influencing the elder abuse by family caregivers among the older adults under hemodialysis. The results of this study can help health policy makers to develop and implement care programs for older people experiencing hemodialysis. Using the SEM method to test the relationship between the studied variables.

## Supplementary Information

**Additional file 1: Model 1.** Standard coefficients of the modified model

## Data Availability

Data generated or analysed during this study are included in this published article and are available from the corresponding author on reasonable request.

## Abbreviations

WHO: World health organization
CKD: Chronic Kidney Disease
HD: Hemodialysis
SEM: Structural Equation Modeling
HCPs: Healthcare professionals
CMIN/DF: Chi-square/degree of freedom ratio
NFI: Normed Fit Index
CFI: Comparative fit index
GFI: Goodness of fit index
SRMR: Standardized root mean squared residual
