# Supplementary figures and images for: Structural equation model of affecting factors on elder abuse to patients under hemodialysis by family caregivers
Source: BMC Geriatr. 2021 Jun 12;21:360. doi: 10.1186/s12877-021-02291-x (PMC8196521; doi:10.1186/s12877-021-02291-x)

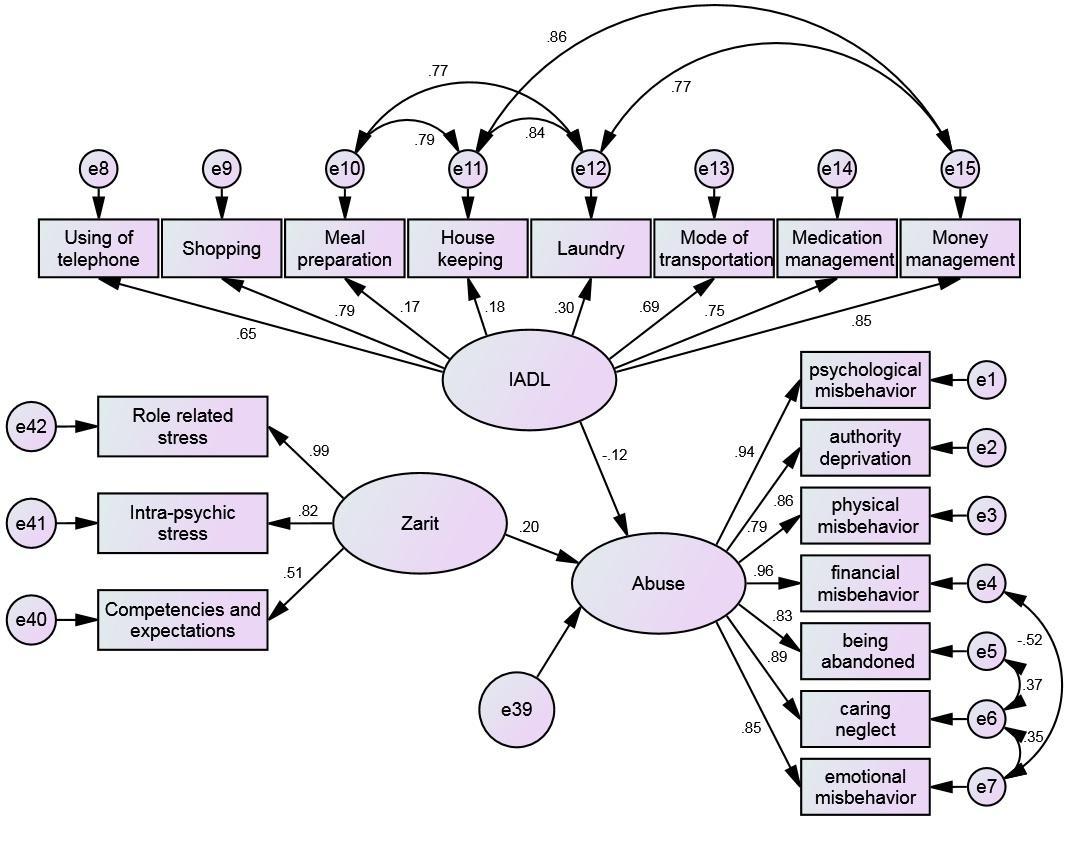


**Model 1. Standard coefficients of the modified model**

Supplement: Supplementary file 1 — Additional file 1: Model 1. Standard coefficients of the modified model [file 12877_2021_2291_MOESM1_ESM.doc]
